# Radiological classification of non-anastomotic biliary strictures after liver transplantation

**DOI:** 10.1007/s00330-026-12515-6

**Published:** 2026-04-17

**Authors:** Chikako Endo, Jules J. G. Slangen, Rianne van Rijn, Iris E. M. de Jong, Hans Blokzijl, Joris Blondeel, Miriam Cortes Cerisuelo, Minneke J. Coenraad, Sarwa Darwish Murad, Michail Doukas, Hasan Eker, Volkert A. L. Huurman, Vincent E. de Meijer, Diethard Monbaliu, Ivo J. Schurink, Wojciech G. Polak, Jeroen de Jonge, Robert J. Porte, Robbert J. de Haas

**Affiliations:** 1 Department of Surgery, Section Hepatobiliary Surgery and Liver Transplantation, University of Groningen, University Medical Center Groningen, Groningen, The Netherlands; 2 Erasmus MC Transplant Institute, Department of Surgery, Division of HPB and Transplant Surgery, Erasmus University Medical Center, Rotterdam, The Netherlands; 3 Department of Radiology, University of Groningen, University Medical Center Groningen, Groningen, The Netherlands; 4 Department of Gastroenterology and Hepatology, University of Groningen, University Medical Center Groningen, Groningen, The Netherlands; 5 Transplantation Research Group, Department of Microbiology, Immunology, and Transplantation, KU Leuven and Department of Abdominal Transplantation Surgery and Coordination, University Hospitals Leuven, Leuven, Belgium; 6 Institute of Liver Studies, Kings College Hospital NHS Foundation Trust, London, United Kingdom; 7 Department of Gastroenterology and Hepatology, Transplant Center, Leiden University Medical Center, Leiden, The Netherlands; 8 Department of Gastroenterology and Hepatology, Erasmus University Medical Center, Rotterdam, The Netherlands; 9 Department of Pathology, Erasmus University Medical Center, Rotterdam, The Netherlands; 10 Department of Transplant Surgery, Ghent University Hospital, Ghent, Belgium; 11 Department of Surgery, Transplant Center, Leiden University Medical Center, Leiden, The Netherlands

**Keywords:** Magnetic resonance imaging, Liver transplant, Biliary tract diseases

## Abstract

**Objectives:**

Due to donor liver shortage, donation after circulatory death (DCD) livers are increasingly used for liver transplantation (LT). However, non-anastomotic biliary strictures (NAS) are more often observed after DCD LT, which can cause serious morbidity. To provide early adequate NAS-treatment, reliable NAS classification is pivotal. Therefore, the current study determined the clinical applicability of two radiological NAS-classification systems, namely the system of Croome and the Groningen system.

**Materials and methods:**

Patients included in the dual hypothermic oxygenated perfusion (DHOPE)-DCD trial (NCT02584283) who underwent LT between January 2016 and July 2019, and in whom per-protocol biliary imaging studies 6 months post-LT were available, were included in the study. NAS severity according to both scoring systems was scored by two independent radiologists, and the correlation for each system with clinical outcomes was made.

**Results:**

In total, 133 patients were included. In our study population, both systems showed good correlation with clinical outcomes, as the highest rates of NAS-related cholangitis and biliary interventions were observed in patients with diffuse necrosis or multifocal progressive disease according to the Croome classification, and likewise in the moderate and severe NAS subgroups in the Groningen classification. Worst 5-year graft and patient survival rates were observed in the case of diffuse necrosis (77% and 80%) or severe NAS (81% and 67%).

**Conclusion:**

Both the radiological NAS classification system of Croome and the Groningen NAS classification system correlate well with clinical outcomes. As the Groningen NAS classification system does not necessitate follow-up imaging, it may be the preferred radiological NAS classification system.

**Key Points:**

***Question*** *Given the potential severity of non-anastomotic biliary strictures after liver transplantation, there is a critical need for adequate radiological classification systems with strong clinical correlations.*

***Findings*** *Both radiological non-anastomotic biliary strictures classification systems of Croome et al and the Groningen system correlated well with clinical outcomes, but no follow-up imaging is needed in the Groningen system.*

***Clinical relevance*** *The Groningen non-anastomotic biliary strictures classification system is easy-to-use, serves as treatment guidance, and provides important prognostic information.*

**Graphical Abstract:**

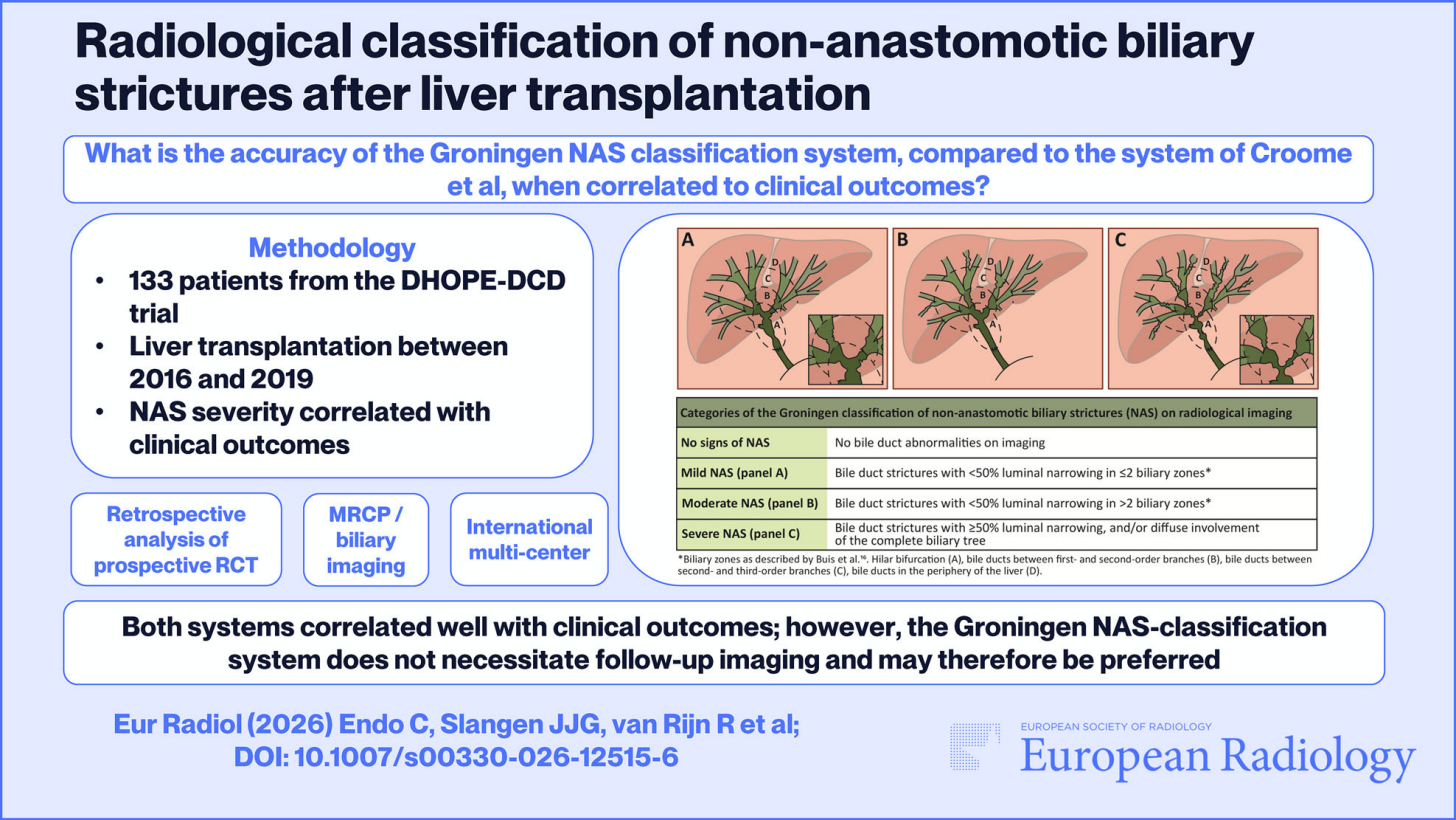

## Introduction

Due to escalating waiting lists for liver transplantation (LT), livers procured from donation after circulatory death (DCD) donors are increasingly used [1]. In some Western countries, DCD LT accounts for almost half of the total number of LTs [2]. However, non-anastomotic biliary strictures (NAS), also known as ischemic type biliary lesions or post-transplant cholangiopathy, associated with ischemia-reperfusion injury, occur more often after DCD liver transplantation, compared to transplantation of livers donated after brain death (DBD) [3–5]. In the literature, the reported incidence of NAS varies between 2.4% and 38%, with most studies focusing on severe cases requiring re-transplantation [6–14]. However, the precise radiological and clinical prevalence of NAS post-LT is much less documented.

The manifestation of NAS following LT can yield significant complications, including recurrent episodes of cholangitis that may necessitate multiple interventions such as endoscopic retrograde cholangiopancreatography (ERCP) or percutaneous transhepatic cholangiodrainage (PTCD). In severe instances, this can even culminate in the need for re-transplantation. Consequently, a comprehensive radiological classification of NAS, coupled with a precise correlation to clinical outcomes, is imperative for optimizing management strategies. In this manner, patients with NAS after LT can be provided with timely and appropriate therapeutic interventions [15]. Currently, several radiological classification systems for NAS are available [16–19], with the most recent being the grading system introduced by Croome et al [15]. In this classification system, four distinct patterns of NAS are delineated, with one pattern also accounting for changes over time (i.e., progression). However, the grading system of Croome et al is based on a relatively heterogeneous study population, in which not all patients underwent routine biliary imaging after LT. At our institution, a more straightforward NAS classification system is employed, comprising three distinct types of NAS, without the requirement for follow-up imaging.

The objective of this study is to determine the accuracy of the NAS classification proposed by Croome et al, alongside our in-house developed NAS classification system (the Groningen classification). We aimed to correlate both systems with clinical outcomes. To achieve this, we analyzed data from patients enrolled in a prospective clinical trial (dual hypothermic oxygenated perfusion (DHOPE)-DCD trial) [20], as these individuals underwent protocol-mandated biliary imaging of the transplanted liver at 6 months post-LT.

## Materials and methods

### Study population

The patient cohort for the current retrospective investigation was extracted from the DHOPE-DCD trial, which included 156 participants across six European transplant centers (NCT02584283). These adult (≥ 18 years old) individuals underwent DCD liver transplantation between January 2016 and July 2019, and a protocolized magnetic resonance cholangiopancreatography (MRCP) was planned at 6 months after transplantation [20]. Patients were eligible for inclusion in the current study if a 6-month MRCP was available or if adequate biliary imaging was performed for clinical reasons before 6 months post-LT. Follow-up images included MRCP, but also other imaging studies of the donor bile ducts, such as cholangiography during ERCP or PTCD. Furthermore, clinical variables, such as the frequency of cholangitis episodes, laboratory values, number of ERCP and PTCD interventions, number of re-LTs, and long-term outcomes (including both graft and patient survival), were systematically collected for each patient.

The research ethics committees at each participating hospital approved the study protocol, and written informed consent was obtained from all participating patients. This retrospective analysis of the previously conducted prospective DHOPE-DCD trial adhered to the guidelines set forth by the code of Good Clinical Practice and the principles outlined in the Declaration of Helsinki.

### Radiological NAS classification

All biliary imaging studies were independently assessed by two abdominal radiologists with expertise in interpreting MRCP images. Both radiologists were blinded to the clinical symptoms, laboratory results, and long-term outcomes of the patients.

**Fig. 1:**
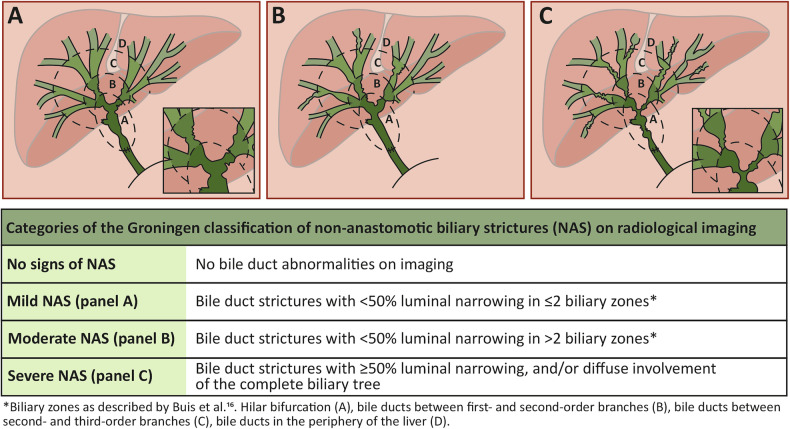
Groningen classification of non-anastomotic biliary strictures after liver transplantation on radiological imaging. Mild NAS is illustrated by panel **a**, moderate NAS by panel **b**, and severe NAS by panel **c**

**Fig. 2:**
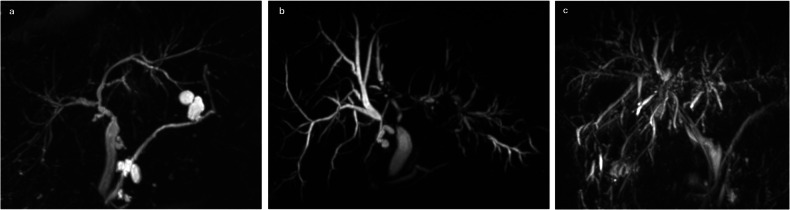
Magnetic resonance cholangiopancreatography (MRCP) images with examples of the various subgroups of the Groningen non-anastomotic biliary strictures (NAS)-classification system; **a** mild NAS; **b** moderate NAS; **c** severe NAS

The severity of radiological NAS was determined by using both the Croome classification system and our in-house developed Groningen classification. In the Croome classification, four distinct patterns of NAS are identified: (1) diffuse necrosis (i.e., severe abnormalities of the entire biliary tree), (2) multifocal progressive (i.e., mild to moderate stenosis of second-order and peripheral ducts progressively worsening over time), (3) confluence dominant (i.e., strictures confined to the biliary confluence that never expand beyond the confluence), and (4) a minor form (i.e., mild radiologic biliary abnormalities consistent with ischemic cholangiopathy) [15]. To ascertain whether the multifocal progressive NAS subtype was present, follow-up imaging studies, when available, were also evaluated. In case of more than one available follow-up imaging study, all studies were evaluated for the presence or absence of multifocal progressive NAS. The Groningen classification is an adapted version of the NAS grading system initially introduced by Buis et al [16], and comprises three subtypes: (1) mild NAS, (2) moderate NAS, and (3) severe NAS. A comprehensive description and schematic examples of each subtype of the Groningen classification are provided in Fig. 1. Examples on MRCP of the subtypes of each NAS-classification system can be found in Fig. 2 and Supplementary Fig. 1.

In cases where the scores provided by the two independent readers differed, a consensus reading was conducted, and a single score for each classification system was assigned.

### Clinical evaluation

The presence of symptomatic NAS was defined by the occurrence of pruritus, jaundice, cholangitis, or abnormal cholestatic laboratory values (exceeding twice the upper limit), or a combination of these, in the context of a patent hepatic artery. The number of ERCP and PTCD procedures performed for the treatment of NAS was recorded up to 5 years post-LT. All interventions performed during the same ERCP or PTCD procedure (e.g., removal of biliary casts, directly followed by balloon dilatation and/or stenting of intrahepatic biliary tracts) were considered one treatment. Clinical cholangitis was diagnosed when fever, accompanied by elevated cholestatic laboratory values, necessitated antibiotic treatment and/or ERCP/PTCD intervention.

### Statistical analysis

Continuous data are reported as median with interquartile range (IQR), unless otherwise specified. Categorical data are expressed as number and percentage. Normality was assessed using the Shapiro–Wilk test. Fisher’s exact test was used for comparisons between groups for categorical variables, while comparisons for continuous variables were conducted using the ANOVA test or independent-samples *t*-test, as appropriate. NAS-related overall patient and graft survival probabilities were depicted using Kaplan–Meier curves, and groups were compared with the log-rank test. *p*-values ≤ 0.05 were deemed statistically significant. Statistical analyses were performed using SPSS software, version 28.0.

## Results

### Study population

**Fig. 3:**
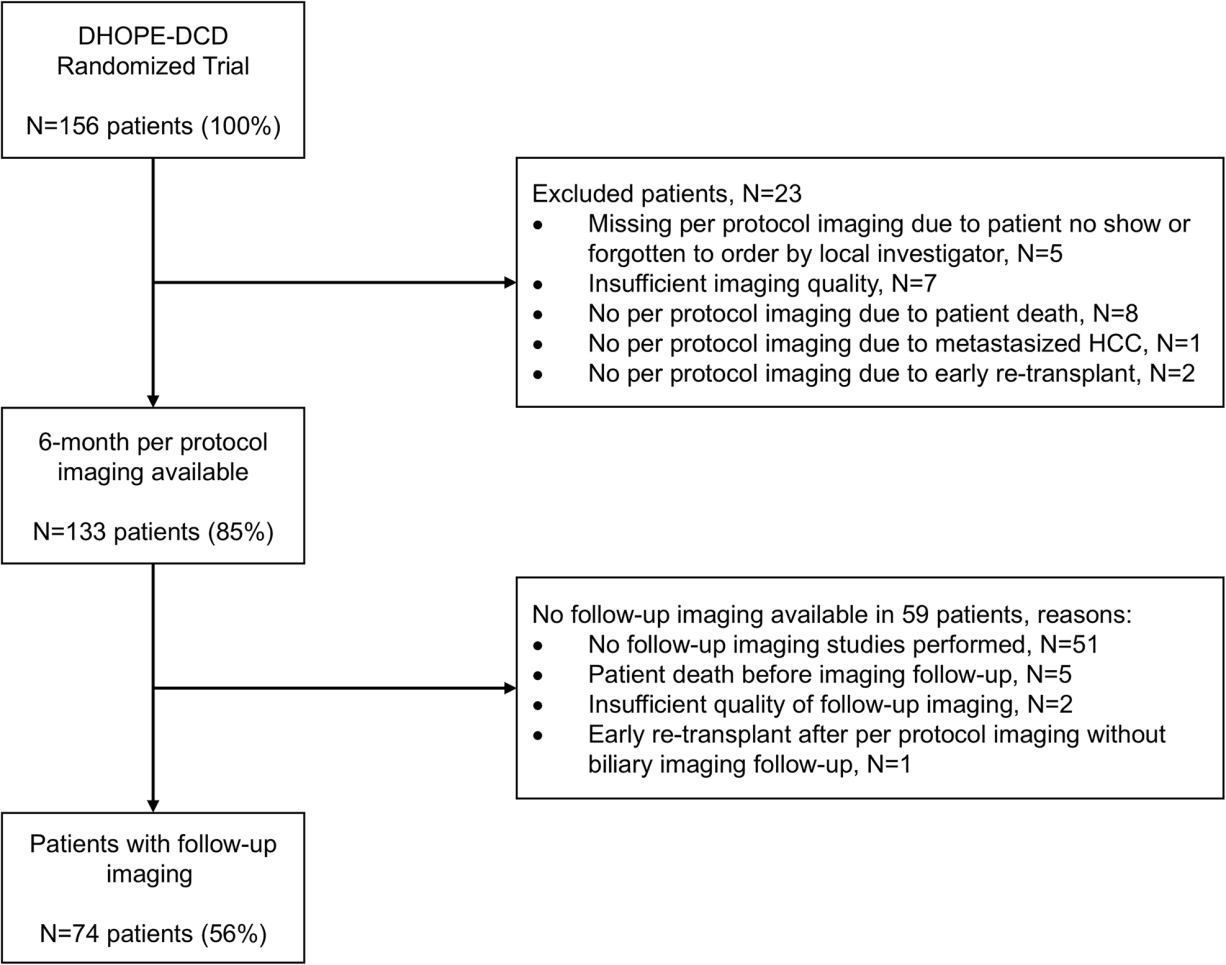
Flowchart study population. DHOPE-DCD, dual hypothermic oxygenated perfusion in ‘donation after cardiac death’ liver grafts; HCC, hepatocellular carcinoma

Between January 2016 and July 2019, a total of 156 patients were enrolled in the DHOPE-DCD trial. Of these, 133 (85%) underwent biliary imaging at or before 6 months after LT. Consequently, these 133 patients were included in the current study (Fig. 3). Of the 133 patients, 118 (89%) underwent per-protocol MRCP, while 15 patients (11%) had cholangiography performed during ERCP or PTCD procedures, or intraoperatively. Standard follow-up biliary imaging was not incorporated into the DHOPE-DCD study protocol and was only performed at the discretion of the attending physicians. Consequently, follow-up biliary imaging studies were available for 74 patients (56%). The reasons for the absence of follow-up imaging are outlined in Fig. 3.

The study cohort comprised 89 men (67%) and 44 women (33%), with a median age at LT of 59 years (IQR: 51–65 years). The most common indication for LT was alcohol-induced liver disease (*N* = 33 (25%)). More detailed recipient characteristics are provided in Table 1, while donor characteristics are outlined in Table 2.

**Table 1:** Recipient characteristics in patients with 6-month per-protocol imaging available (*N* = 133)

Variable		
Median age at transplant, years (IQR)		59 (51–65)
Sex (male/female)		89 (67%)/44 (33%)
Median BMI, kg/m^2^ (IQR)		27 (23–30)
LT indication/underlying liver disease		
	Viral hepatitis	4 (3%)
	Alcohol-induced liver disease	33 (25%)
	MASLD	16 (12%)
	Primary biliary cholangitis	12 (9%)
	Primary sclerosing cholangitis	21 (16%)
	Metabolic liver disease	5 (4%)
	HCC	21 (16%)
	Cryptogenic	5 (4%)
	Other	16 (12%)
Median MELD score (IQR)		15 (10–20)
Type of biliary anastomosis		
	Duct-to-duct	119 (90%)
	Hepatico-jejunostomy	14 (11%)

*BMI* body mass index, *HCC* hepatocellular carcinoma, *IQR* interquartile range, *LT* liver transplant, *MASLD* metabolic dysfunction-associated steatotic liver disease, *MELD* model for end-stage liver disease

**Table 2:** Donor characteristics in patients with 6-month per-protocol imaging available (*N* = 133)

Variable	
Median age at transplant, years (IQR)	51 (40–57)
Sex (male/female)	91 (68%)/42 (32%)
Median BMI, kg/m^2^ (IQR)	25 (22–27)
Median total DWIT, minutes (IQR)	11 (8–14)
Median total CIT, minutes (IQR)	382 (332–441)

*BMI* body mass index, *CIT* cold ischemia time, *DWIT* donor warm ischemia time, *IQR* interquartile range

### Radiological NAS classification according to the Croome classification and correlation with clinical outcome

According to the NAS classification system described by Croome et al, 45 (34%) patients exhibited no signs of NAS, 12 (9%) were classified as having diffuse necrosis, 18 (14%) had multifocal progressive NAS, 27 (20%) belonged to the confluence dominant NAS subgroup, and 31 (23%) patients presented with a minor form of NAS (Table 3).

**Table 3:** Overview of outcomes per radiological NAS subgroup introduced by Croome et al (*N* = 133)

		Normal (*N* = 45)	Diffuse necrosis (*N* = 12)	Multifocal progressive (*N* = 18)	Confluence dominant (*N* = 27)	Minor form (*N* = 31)	*p*-value	Overall (*N* = 133)
Median period between initial LT and symptomatic NAS diagnosis, days (IQR)		-	136 (71–316)	137 (78–406)	193 (88–307)	187 (43–397)	0.73^a^	147 (76–311)
Symptomatic NAS (%)		1 (2%)	8 (67%)	7 (39%)	8 (30%)	7 (23%)	< 0.001^b^	31 (23%)
Total number of ERCPs/PTCDs for NAS							< 0.001^b^	
	0 ≥ 1	45 (100%) 0 (0%)	4 (33%) 8 (67%)	14 (78%) 4 (22%)	20 (74%) 7 (26%)	29 (94%) 2 (7%)		112 (84%) 21 (16%)
Total number of NAS-related cholangitis episodes							< 0.001^b^	
	0 ≥ 1	44 (98%) 1 (2%)	6 (50%) 6 (50%)	12 (67%) 6 (33%)	22 (82%) 5 (19%)	28 (90%) 3 (10%)		112 (84%) 21 (16%)
NAS-related re-LT (%)		0 (0%)	2 (17%)	4 (22%)	1 (4%)	1 (3%)	0.003^b^	8 (6%)
Period between initial LT and NAS-related re-LT		-	Patient 1: 724 days Patient 2: 1079 days	Patient 1: 124 days Patient 2: 390 days Patient 3: 425 days Patient 4: 872 days	Patient 1: 807 days	Patient 1: 1297 days	-	-
NAS-related actual death (%)		0 (0%)	2 (17%)	1 (6%)	3 (11%)	0 (0%)	0.01^b^	6 (5%)
Period between initial LT and NAS-related death		-	Patient 1: 359 days Patient 2: 732 days	Patient 1: 1220 days	Patient 1: 261 days Patient 2: 600 days Patient 3: 1422 days	-	-	-
Median NAS-related graft survival, days (IQR)		1681 (1315–1919)	1008 (578–1966)	1579 (771–1982)	1678 (600–2013)	1836 (1548–2218)	0.004^c^	1678 (1216–2017)
Median NAS-related overall patient survival, days (IQR)		1681 (1315–1919)	1299 (580–1966)	1579 (722–1982)	1678 (365–2013)	1836 (1548–2218)	0.02^c^	1678 (1259–2017)

*ERCP* endoscopic retrograde cholangiopancreatography, *IQR* interquartile range, *LT* liver transplant, *NA* not applicable, *NAS* non-anastomotic biliary strictures, *PTCD* percutaneous transhepatic cholangiodrainage, *re-LT* repeat liver transplant

^a^ ANOVA test

^b^ Fisher’s exact test

^c^ Log-rank test

When considering the total number of NAS-related cholangitis episodes, a significantly greater proportion of patients with diffuse necrosis or multifocal progressive NAS experienced more than one episode, compared to those classified as normal or with minor NAS (50% and 33% versus 2% and 10%, respectively; *p* < 0.001). Similarly, a higher percentage of patients underwent more than one ERCP or PTCD procedure when classified with diffuse necrosis or multifocal progressive NAS, in contrast to those with the minor form of NAS or without any signs of NAS (67% and 22% versus 7% and 0%, respectively; *p* < 0.001).

**Fig. 4:**
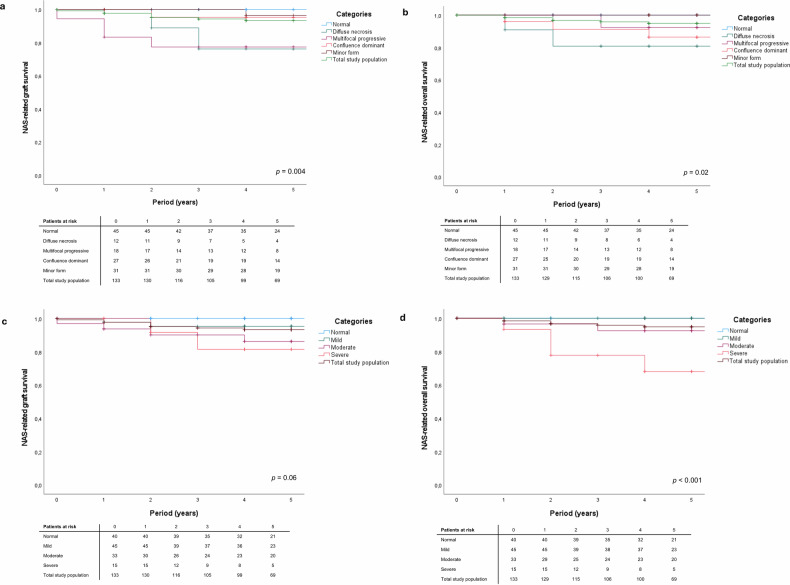
Kaplan–Meier curves displaying non-anastomotic biliary strictures (NAS)-related graft and overall survival rates for each NAS-classification system. **a**, **b** Stratified by the NAS-classification system introduced by Croome et al; **c**, **d** Stratified by the Groningen NAS-classification system

Repeat LT was more frequently required in patients with diffuse necrosis or multifocal progressive NAS, compared to those with minor or no signs of NAS (17% and 22% versus 3% and 0%, respectively; *p* = 0.003). As a result, NAS-related 5-year graft survival rates were 100% and 96% in patients with no or minor NAS, respectively, as opposed to 77% in those with diffuse necrosis or multifocal progressive NAS (*p* = 0.004; Fig. 4a). NAS-related overall patient survival was highest in patients with no or minimal signs of NAS (5-year overall patient survival was 100% in both groups, compared to 80% in the diffuse necrosis group; *p* = 0.02; Fig. 4b). Further details can be found in Table 3.

### Radiological NAS classification by using the Groningen system and correlation with clinical outcomes

Using the Groningen classification system, 40 (30%) patients were considered as normal, 45 (34%) as having mild NAS, 33 (25%) as having moderate NAS, and 15 patients (11%) as having severe NAS (Table 4).

**Table 4:** Overview of outcomes per radiological NAS subgroup according to the Groningen classification (*N* = 133)

		Normal (*N* = 40)	Mild (*N* = 45)	Moderate (*N* = 33)	Severe (*N* = 15)	*p*-value	Overall (*N* = 133)
**At 6-month per-protocol imaging moment**							
Median period between initial LT and symptomatic NAS diagnosis, days (IQR)		970 (NA)	100 (58–249)	94 (78–210)	270 (86–369)	< 0.001^a^	147 (76–311)
Symptomatic NAS (%)		2 (5%)	9 (20%)	11 (33%)	9 (60%)	< 0.001^b^	31 (23%)
Total number of ERCPs/PTCDs for NAS						< 0.001^b^	
	0 ≥ 1	39 (98%) 1 (2%)	39 (87%) 6 (13%)	28 (85%) 5 (15%)	6 (40%) 9 (60%)		112 (84%) 21 (16%)
Total number of NAS-related cholangitis episodes						0.01^b^	
	0 ≥ 1	38 (95%) 2 (5%)	39 (87%) 6 (13%)	26 (79%) 7 (21%)	9 (60%) 6 (40%)		112 (84%) 21 (16%)
NAS-related re-LT (%)		0 (0%)	2 (4%)	4 (12%)	2 (13%)	0.05^b^	8 (6%)
Period between initial LT and NAS-related re-LT		-	Patient 1: 425 days Patient 2: 724 days	Patient 1: 124 days Patient 2: 390 days Patient 3: 807 days Patient 4: 1297 days	Patient 1: 872 days Patient 2: 1079 days	-	-
NAS-related actual death (%)		0 (0%)	0 (0%)	2 (6%)	4 (27%)	< 0.001b	6 (5%)
Period between initial LT and NAS-related death		-	-	Patient 1: 261 days Patient 2: 1220 days	Patient 1: 359 days Patient 2: 600 days Patient 3: 732 days Patient 4: 1422 days	-	-
Median NAS-related graft survival, days (IQR)		1667 (1331–1856)	1659 (1467–2033)	1890 (757–2185)	1422 (600–1836)	0.06^c^	1678 (1216–2017)
Median NAS-related overall patient survival, days (IQR)		1667 (1331–1856)	1659 (1516–2033)	1890 (618–2185)	1422 (600–1836)	< 0.001^c^	1678 (1259–2017)

*ERCP* endoscopic retrograde cholangiopancreatography, *IQR* interquartile range, *LT* liver transplant, *NA* not applicable, *NAS* non-anastomotic biliary strictures, *PTCD* percutaneous transhepatic cholangiodrainage, *re-LT* repeat liver transplant

^a^ ANOVA test

^b^ Fisher’s exact test

^c^ Log-rank test

The proportion of patients with more than one NAS-related cholangitis episode was significantly higher in the subgroup classified as severe NAS, compared to those with no or only mild signs of NAS (40% in the severe NAS subgroup, compared to 5% and 13% in the normal and mild subgroups, respectively; *p* = 0.01). Similarly, the need for one or more ERCP or PTCD procedures for NAS was highest in patients with severe NAS (60% versus 2% and 13% in the normal and mild subgroups, respectively; *p* < 0.001).

The rate of repeat LTs was highest in patients with moderate or severe NAS (12% and 13%, respectively), compared to 0% and 4% in the normal and mild subgroups (*p* = 0.05). As a result, the 5-year NAS-related graft survival rates were 86% and 81% in the moderate and severe subgroups, respectively, compared to 100% and 95% in the normal and mild NAS subgroups (*p* = 0.06; Fig. 4c). The highest 5-year NAS-related overall survival rates were observed in patients with no or only mild signs of NAS (100% in both subgroups), compared to 93% and 67% in those with moderate or severe NAS, respectively (*p* < 0.001; Fig. 4d).

## Discussion

Given the potential severity of NAS after LT, which may sometimes necessitate repeat LT, there is a critical need for robust NAS radiological classification systems with strong clinical correlations [21]. These systems are essential for providing timely and optimal treatment to patients and to precisely inform patients regarding their prognosis. Therefore, the aim of the present study was to compare the recently introduced NAS classification by Croome et al to our in-house developed Groningen NAS classification, by correlating both systems with clinical outcomes. To ensure the reliability of the results, both classification systems were applied to patients from the DHOPE-DCD trial, all of whom underwent per-protocol biliary imaging of the transplant liver 6 months after LT, regardless of the presence or absence of clinical signs of NAS.

When applying both NAS classification systems to our study population, both systems demonstrated a strong correlation with clinical outcomes. Specifically, in both classifications, subgroups with more severe NAS were associated with poorer short-term and long-term outcomes. The highest rates of NAS-related cholangitis episodes and greatest frequencies of biliary interventions were observed in patients with diffuse necrosis or multifocal progressive disease in the Croome classification, as well as the moderate or severe NAS subgroups of the Groningen classification. Similarly, the poorest 5-year NAS-related graft and overall survival rates were observed in patients with diffuse necrosis according to the Croome classification, and in those with severe NAS in the Groningen classification (77% and 80% versus 81% and 67%, respectively).

Several radiological classification systems for NAS after LT have been proposed in the literature [15–19]. However, many of these systems are relatively outdated, particularly when considering the significant advancements made over the past decade in both imaging quality and treatment techniques. Among these systems, the most recent was introduced by Croome et al in 2022 [15]. To ensure the most reliable results regarding the accuracy of our in-house developed Groningen NAS classification system, we compared it with the Croome classification. In doing so, we believe that the Groningen classification has been evaluated against the most current and relevant classification system available in the literature, thus facilitating optimal translation to modern clinical practice. More specifically, its strong correlation with clinical outcomes enables the Groningen NAS classification, when combined with clinical judgment, to guide treatment decisions and offer patients prognostic information such as expected (NAS-related) graft survival.

The radiological NAS classification system introduced by Croome et al in 2022 was based on a relatively heterogeneous patient population from several centers [15]. This multicenter retrospective study included 770 patients who underwent DCD LT between 1999 and 2020. In two of the centers, per-protocol cholangiograms through a biliary catheter were obtained on postoperative days 3 and 21, with follow-up biliary imaging performed only when clinically indicated. In the third center, biliary imaging was conducted based on clinical presentation. Consequently, routine biliary imaging was only performed in part of the study population, and it was done shortly after LT. All other imaging studies were conducted based on clinical indications. In contrast, the current study applied both radiological NAS classification systems (i.e., the system introduced by Croome et al and the Groningen system) to a patient population derived from the prospective DHOPE-DCD trial [20] in which all patients underwent per-protocol biliary imaging 6 months after LT, regardless of clinical symptoms. This approach resulted in a homogeneous study population, providing a unique opportunity to compare both radiological NAS classification systems independent of clinical presentation. Furthermore, in the vast majority of our study population, biliary imaging was performed using MRCP, which is currently regarded as the primary imaging modality for detecting biliary complications after LT, with a reported sensitivity of 98–99% and specificity of 94–96% [22]. Therefore, the results of both classification systems presented in this study are based on recent data obtained under optimal conditions, offering the most reliable reflection of their clinical value.

In the Croome classification, four distinct NAS subtypes were identified: a minor form, diffuse necrosis, multifocal progressive NAS, and a confluence dominant form [15]. In their study, Croome et al observed that patients with the diffuse necrosis NAS subtype had the worst outcomes in terms of 5-year graft survival, followed by those in the multifocal progressive NAS subgroup (0% and 20.8%, respectively). In contrast, the 5-year graft survival rates for the confluence dominant and minor NAS subgroups were reported to be 78.1% and 80.0%, respectively [15]. When applying the Croome classification to our study population, the 5-year graft survival rates were 77% for patients with diffuse necrosis or multifocal progressive NAS, and 95% and 96% for those with confluence dominant or minor NAS, respectively. The better long-term outcomes observed in our study may be attributed to the more homogeneous study population in the current study, as well as the use of more advanced imaging and surgical techniques. Notably, our cohort consisted of patients transplanted between 2016 and 2019, in contrast to the 1999–2020 period in the Croome et al study. Another limitation of the NAS classification system proposed by Croome et al is the requirement for follow-up biliary imaging, as progressive disease can only be assessed through serial imaging studies. On the other hand, the Groningen NAS classification system relies on a single imaging study, eliminating the need for follow-up biliary imaging, which improves its applicability in daily clinical practice. When follow-up imaging becomes available for clinical reasons, NAS classification can be adjusted as NAS progression is sometimes a dynamic process.

The retrospective analysis of the current study may be considered a limitation. However, the fact that the study population was derived from the prospective DHOPE-DCD trial, which resulted in a homogeneous cohort where all patients underwent per-protocol biliary imaging 6 months after DCD LT regardless of clinical symptoms, suggests that the retrospective nature does not substantially impact the reliability of the results. In line with this, the retrospective analysis of clinical outcomes does not seem to be of significant impact on the results. Another potential limitation of our study is the inherent subjectivity involved in radiologically classifying NAS. However, all imaging studies were independently reviewed by two abdominal radiologists with extensive experience in liver transplant imaging, and in cases of conflicting interpretations, a final diagnosis was reached through consensus reading. In our view, this approach represents the most objective method for radiologically classifying NAS. The lack of histopathological proof of the presence of NAS can be considered a limitation; however, our study reflects daily clinical practice combining radiological bile duct abnormalities and clinical judgment to determine the most optimal treatment, without the need for histopathological proof of NAS. Looking ahead, besides the need for external validation of the Groningen NAS classification system, there may be a role for artificial intelligence techniques in enhancing the objectivity and accuracy of NAS classification.

In conclusion, both the radiological NAS classification system introduced by Croome et al and the Groningen radiological NAS classification system demonstrated adequate correlation with clinical outcomes. However, the system of Croome et al requires follow-up imaging, whereas the Groningen NAS classification system does not, making the latter more suitable for prospective use in predicting clinical outcomes. Therefore, the Groningen classification appears to be an easy-to-use radiological NAS classification system with strong clinical correlation.

## Supplementary information

Supplementary information

## Abbreviations

DCD: Donation after circulatory death
DHOPE: Dual hypothermic oxygenated perfusion
ERCP: Endoscopic retrograde cholangiopancreatography
IQR: Interquartile range
LT: Liver transplantation
MRCP: Magnetic resonance cholangiopancreatography
NAS: Non-anastomotic biliary strictures
PTCD: Percutaneous transhepatic cholangiodrainage
