# Supplementary material for: Radiological classification of non-anastomotic biliary strictures after liver transplantation
Source: Eur Radiol. 2026 Apr 17;36(8):6535–45. doi: 10.1007/s00330-026-12515-6 (PMC13342394; doi:10.1007/s00330-026-12515-6)
Supplement: Supplementary file 1 — Supplementary information [file 330_2026_12515_MOESM1_ESM.pdf]

Radiological Classification of Non-anastomotic Biliary Strictures after Liver Transplantation

ELECTRONIC SUPPLEMENTARY MATERIAL

Supplementary Table 1

Supplementary table 1: Recipient characteristics in patients with and without 6-month per protocol imaging available (N=156)

**Variable**	**Six-month per protocol imaging available (N=133)**	**Six-month per protocol imaging not available (N=23)**	***p***
Median age at transplant, years (IQR)	59 (51-65)	62 (58-69)	0.27[a]
Sex (male / female)	89 (67%) / 44 (33%)	18 (78%) / 5 (22%)	0.34[b]
Median BMI, kg/m^2 (IQR)	27 (23-30)	28 (24-30)	0.30[a]
LT indication / underlying liver disease			
Viral hepatitis	4 (3%)	3 (13%)	0.07[b]
Alcoholic-induced liver disease	33 (25%)	8 (35%)	0.32[b]
MASLD	16 (12%)	5 (22%)	0.20[b]
Primary biliary cholangitis	12 (9%)	1 (4%)	0.69[b]
Primary sclerosing cholangitis	21 (16%)	1 (4%)	0.32[b]
Metabolic liver disease	5 (4%)	1 (4%)	0.62[b]
HCC	21 (16%)	3 (13%)	0.51[b]
Cryptogenic	5 (4%)	0 (0%)	0.45[b]
Other	16 (12%)	0 (0%)	0.12[b]
Median MELD score (IQR)	15 (10-20)	13 (8-20)	0.71[a]

[a] independent-samples t test. [b] Fisher's exact test.
Abbreviations: BMI: body mass index; HCC: hepatocellular carcinoma; IQR: interquartile range; LT: liver transplant; MASLD: metabolic dysfunction-associated steatotic liver disease; MELD: model for end-stage liver disease.

Supplementary Figure 1. Magnetic resonance cholangio-pancreatography (MRCP) images with examples of the subgroups of the non-anastomotic biliary strictures (NAS)-classification system introduced by Croome et al.; **(a)**: diffuse necrosis; **(b)**: multifocal progressive, first MRCP; **(c)**: multifocal progressive, MRCP during follow-up; **(d)**: confluence dominant; **(e)**: minor form.

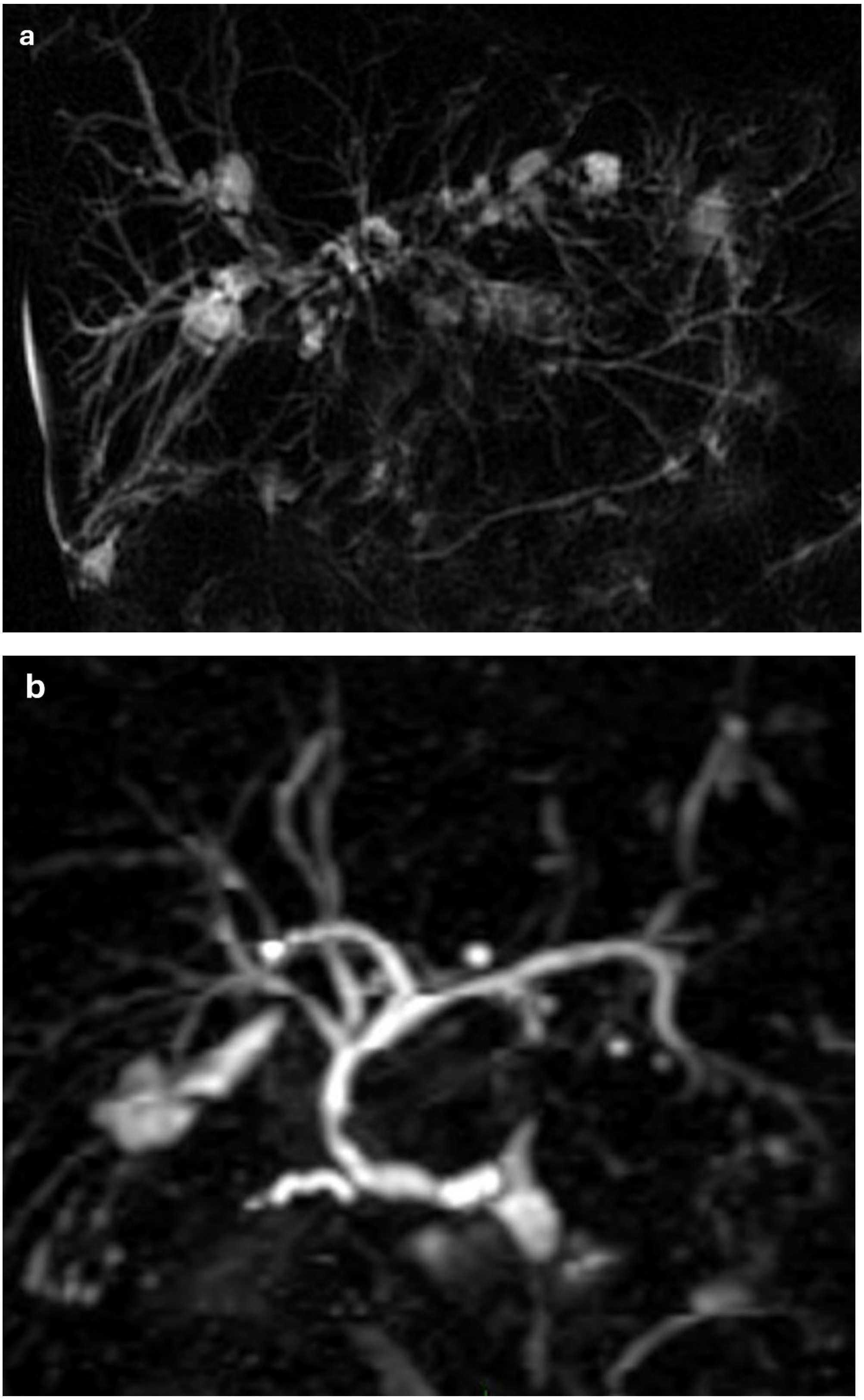

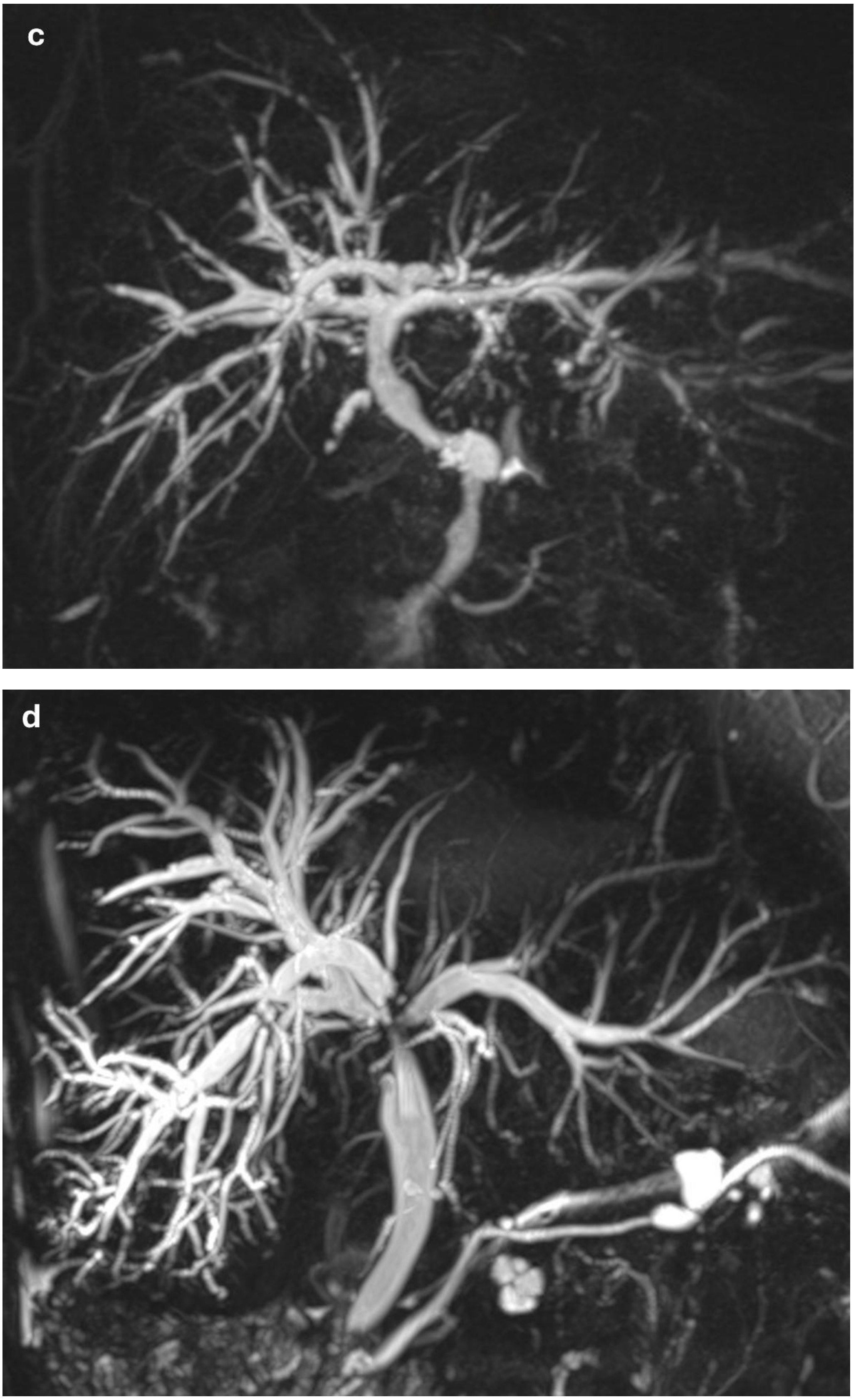
c
d

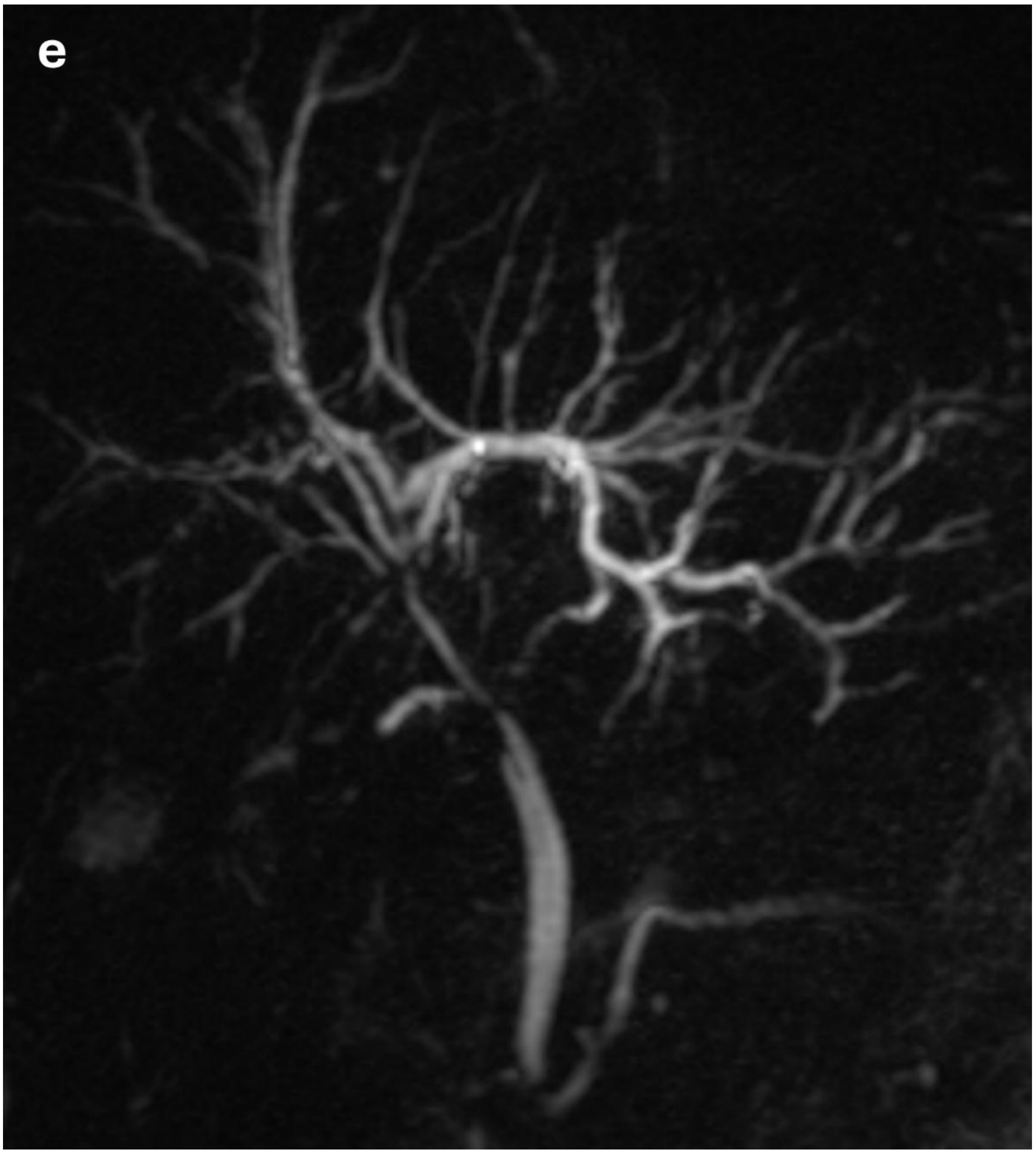
e
